# Twinning in SnO_2_-based ceramics doped with CoO- and Nb_2_O_5_: morphology of multiple twins revealed by electron backscatter diffraction

**DOI:** 10.1107/S2052520620010264

**Published:** 2020-09-15

**Authors:** José Alberto Padrón-Navarta, Fabrice Barou, Nina Daneu

**Affiliations:** a Géosciences Montpellier, Univ. Montpellier, CNRS and Univ. des Antilles, Montpellier, France; b Department for Advanced Materials, Jožef Stefan Institute, Jamova cesta 39, Ljubljana, 1000, Slovenia

**Keywords:** cassiterite, cyclic twins, coplanar twins, alternating twins, nucleation twinning, rutile-type structure, automated twin identification

## Abstract

Electron backscatter diffraction (EBSD) was used for the analysis of multiple cyclic twins in cassiterite (SnO_2_), which form during sintering of SnO_2_ with small additions of CoO and Nb_2_O_5_. Grain misorientation analysis has shown that about one third of all grains contain {101} twin boundaries (TBs). The majority of these grains are contact twins, whereas a small fraction of grains are multiple, mainly cyclic twins. A procedure was developed in *MTEX* [Bachmann, Hielscher & Schaeben (2010). *Solid State Phenom.* **160**, 63–88 ▸] for automated identification of crystallographically different types of cyclic twins and found two main types: coplanar twins composed of three or four domains with a common [010] axis and alternating twins composed of three to seven domains oriented along the [111] axis. Both types of cyclic twins have a characteristic common origin (nucleus) of all TBs, which is positioned eccentric relative to the grain section and the cycle is closed with a shorter non-crystallographic contact between the first and the last twin domain. The morphology of cyclic twins suggests that they form by nucleation in the initial stages of grain growth. The average size of twinned grains increases with the number of twin domains indicating the influence of TBs formation on the growth of composite grains.

## Abstract 2

The *MTEX* procedure for electron backscatter diffraction data treatment allows automated detection of cyclic twins with coplanar and alternating morphology in sintered cassiterite-based ceramics.

## 1. Introduction   

The sintering of SnO_2_ (cassiterite) with small amounts of Co- and Nb-oxides results in the development of a polycrystalline ceramic microstructure composed of randomly oriented cassiterite grains with a fairly large fraction of grains containing {101} twin boundaries (TBs) (Tominc *et al.*, 2018 ▸). In cross-sections, most of the twinned grains are simple contact twins, but multiple (mostly cyclic) twins with up to five domains were observed. In our previous study we focused on the functional properties of the ceramics and densification in relation to the specific charge compensation mechanisms in cassiterite with the addition of aliovalent dopants, whereas questions related to the crystallography of cyclic twins and reasons for their formation remained open.

Cassiterite crystallizes in the rutile-type structure with tetragonal symmetry (point group 4/*m*2/*m*2/*m*; centrosymmetric), where twinning on {101} planes is common (*e.g.* Hahn & Klapper, 2006 ▸). Reflection twins across the (101) plane in rutile-type structures are known as elbow or knee twins; they occur in both of the most common minerals with the rutile-type structure, rutile (TiO_2_) and cassiterite (SnO_2_) (Nespolo & Souvignier, 2015 ▸). Due to the four sets of equivalent {101} reflection planes (tetragonal prisms), different multiple twinning combinations are possible; they have been thoroughly described in rutile (Hahn & Klapper, 2006 ▸). One of the possibilities is random twinning and formation of branched twinned structures in 3D (Jordan *et al.*, 2016 ▸). This type of multiple twin forms by self-assembly during unconstrained growth in three dimensions, *e.g*. by exsolution from saturated hydro­thermal solutions. Another configuration of multiple twins are 2D intergrowths, where all domains intersect at ∼60° and are oriented along a common crystallographic axis. In rutile, such reticulated (or geniculated) intergrowths are known as sagenite, their formation is related to epitaxial growth on a structurally related substrate (epitaxial twinning) or oriented recrystallization of a structurally related precursor (topotaxial twinning) like ilmenite (FeTiO_3_) (Armbruster, 1981 ▸; Force *et al.*, 1996 ▸; Rečnik *et al.*, 2015 ▸). In sagenite networks, all domains have a common [010] axis and the contacts between the domains are either non-crystallographic contacts at 60°or 120° (Force *et al.*, 1996 ▸; Stanković *et al.*, 2015 ▸) or TBs along {101} or {301} planes (Rečnik *et al.*, 2015 ▸). Sagenite intergrowths are characteristic of rutile, whereas in cassiterite they have not been reported. An even more intriguing type of multiple twin corresponds to cyclic twins, which form closed structures with nonparallel twin contacts (Hofmeister, 2004 ▸). The number of twin domains (*N*) is limited by the angle (α) between the two subsequent TBs (*N* = 360°/α). If *N* is not an integer, the cyclic twin is closed by a non-crystallographic contact (NCC). Fully developed coplanar and alternating cyclic twins are common in rutile, but are not typical for cassiterite. Two types of cyclic twins are found in rutile: coplanar with a common [010] axis and alternating with a common [111] axis (Hahn & Klapper, 2006 ▸). However, the mechanisms leading to the formation of cyclic twins are not yet fully understood.

Electron backscatter diffraction (EBSD) mapping is a powerful technique of electron microscopy and enables the determination of absolute orientation of grains and crystal domains in polished samples of polycrystalline ceramics, alloys, and mineralogical and petrological cross-sections (*e.g.* Schwarzer *et al.*, 2009 ▸). The information about crystal orientation is a valuable additional component in classical microstructural analysis, where average grain size, distribution of grain sizes, morphology of grains and similar are the usual parameters of quantitative microstructure evaluation. EBSD in combination with chemical composition analysis using spectroscopic techniques such as energy-dispersive X-ray spectroscopy (EDXS) or electron probe microanalyzer (EPMA) gives a complete picture of the microstructural characteristics of the sample, thus allowing a more in-depth interpretation of its functional properties. EBSD mapping has also become a powerful tool for the characterization of complex twins (Keshavarz & Barnett, 2006 ▸; Beyerlein *et al.*, 2010 ▸). EBSD mapping can cover large surfaces with high spatial resolution (up to few hundreds of nanometres in field-emission scanning electron microscopes), thus providing a statistical mean for identifying the existence and the proportion of different TBs, in addition to the spatial distribution and grain size of twinned grains (*e.g*. Humphreys, 2004 ▸). The identification of TBs using EBSD data relies on the analysis of the crystallographic misorientation along grain boundaries (GB), which is typically described by a rotation axis and a rotation angle. TBs are special boundaries that separate domains of the same phase and are defined by a constant rotation angle and a low-index crystallographic direction. Similarly, epitaxial contacts can be defined when the GB separates two different phases and the misorientation follows a constant rotation angle and a particular crystallographic rotation axis. In both cases, the rotation axis and rotation angle span a 3D parameter space known as misorientation space, which is constrained by the point-group symmetry of the phase or the two point-group symmetries of the two different phases in contact for the case of topotaxy/epitaxy (Krakow *et al.*, 2017 ▸). Routinely EBSD analyses (2D EBSD) only have access to the intersection of any type of grain boundary (GB), including TBs, with the sample section and therefore can only give some constrains (based on the likelihood of sectioning) on the orientation of the GB/TB (Marquardt *et al.*, 2015 ▸), although the morphology of the intersection (straight/curved) is already indicative of the existence or not of low crystallographic index planes. Coupling EBSD mapping with complementary TEM analyses is a powerful tool to get access to the nature of the TBs and ultimately to investigate their formation mechanisms. Moreover, EBSD mapping, in combination with advanced data treatment toolboxes (*e.g. *MTEX**; Hielscher & Schaeben, 2008 ▸; Bachmann *et al.*, 2010 ▸; Mainprice *et al.*, 2014 ▸), is particularly suitable for characterizing complex twinned crystals such as cyclic twins which can be challenging in terms of sample preparation for TEM.

In this work, we applied EBSD mapping for the analysis of twinned grains that frequently occur in SnO_2_-based ceramics co-doped with Co- and Nb-oxides (Tominc *et al.*, 2018 ▸). In addition to untwinned grains and contact twins we were able to automatically classify different types of cyclic and non-cyclic (branched) multiple twins based on the identification of the axis common to all domains forming the twinned aggregate.

## 2. Methods   

### 2.1. Sample preparation   

For our analyses we used samples prepared by the conventional solid-state ceramics processing route as reported previously (Tominc *et al.*, 2018 ▸). The samples were prepared by homogenization of SnO_2_ powder (Alfa Aesar, 99.9%, nanopowder) with the addition of 1 mol% CoO (Alfa Aesar, 95%) and 1 mol% Nb_2_O_5_ (Merck, 99%) in absolute ethanol. The homogenized mixtures were dried, pressed into pellets and sintered at 1430°C for 5 h in air.

### 2.2. EBSD analyses   

Samples for microstructural EBSD analyses were prepared by polishing the ceramic’s cross-sections down to 0.25 µm with diamond paste and subsequent annealing (thermal etching) at 1250°C for 15 min. The heat treatment of the surface resulted in removal (recrystallization) of the amorphous surface layer yielding EBSD patterns of excellent quality with high contrast Kikuchi patterns and a high indexing rate (95%), without the need of further chemical polishing with colloidal silica. EBSD analyses were performed using a dedicated instrument, CamScan X500FE Crystal Probe with a 70° inclined column optimized for EBSD and an field-emission electron gun allowing high-resolution spatial analyses (Géosciences Montpellier). The EBSD patterns were recorded with a CCD HKL Nordlys Nano (Oxford Instruments) camera in uncoated samples at 20 kV accelerating voltage and a working distance of 25 mm. The patterns were indexed automatically using *AZtechHKL* software package from Oxford Instruments. Three areas (Areas 1, 2 and 3) were mapped with a step size of 250 nm covering an area of 249.5 µm × 197.0 µm. Grain reconstruction and grain misorientation boundaries were determined using *MTEX* open source toolbox (https://doi.org/10.4028/www.scientific.net/SSP.160.63) for MATLAB (Hielscher & Schaeben, 2008 ▸; Mainprice *et al.*, 2014 ▸). The recent inverse pole figure (IPF) coloring scheme developed by Nolze & Hielscher (2016 ▸) was used for orientation mapping coloring.

### 2.3. Reconstruction of multiple twin models   

Schematic 3D models of multiple twins with different crystallographic configurations were reconstructed in **VESTA** (Momma & Izumi, 2011 ▸) by using the multiple-phase data option. Absolute orientation for each of the domains of a multiple twin was obtained by expressing in Miller notation the orientation of two perpendicular reference vectors based on the orientation data for each domain obtained from EBSD maps (recorded as Euler angles). In *VESTA*, the orientation of a crystal or crystal domain is given by the out-of-plane (projection) vector **Z** = (*u*,*v*,*w*) and the north direction as the pole of the (*hkl*) in-plane, *i.e.* the (upward) vector **Y** = (*h*,*k*,*l*). A Matlab script for the conversion of Euler angles obtained from EBSD maps to vectors for *VESTA* is given in the supporting information. After inserting the orientation data for all domains into the *VESTA* file, the domains were cut along the proper {101} contact planes common to the neighboring domains. Finally, relative positioning of the domains was done manually to obtain a model with all domains in contact. Each of the domains was colored to match the EBSD colors in order to facilitate the comparison between the 3D twin model and the grain in the 2D cross-section.

## 3. Results   

### 3.1. General characterization of the microstructures   

**Figure 1:**
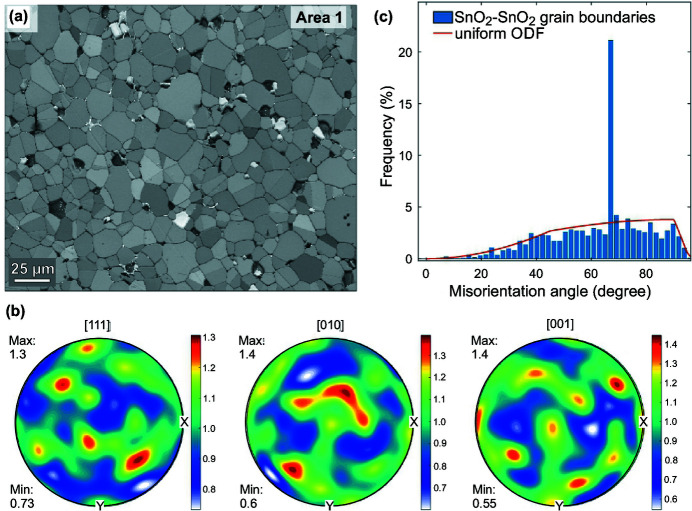
(*a*) Band contrast image of Area 1 shows typical dense microstructure of the polycrystalline ceramics. (*b*) Orientation distribution function plotted as pole figures (mean orientation per grain) for Area 1 showing nearly random orientation of grains, upper hemisphere, contouring refers to multiple of uniform distribution (m.u.d.), maximum and minimum m.u.d. values for each pole figure are indicated. (*c*) Histogram of misorientation angle (data for all three Areas included) across the GBs showing a distribution close to a uniform orientation distribution function (ODF) except for a peak at 67.8°C corresponding to TBs compatible with traces of {101} twin planes.

Fig. 1 ▸(*a*) is a representative band contrast image of the SnO_2_-based ceramics doped with 1 mol% CoO and 1 mol% of Nb_2_O_5_ after sintering at 1430°C (see Fig. S1 for band contrast images of Areas 2 and 3). It shows typical microstructure of the dense polycrystalline ceramics composed of cassiterite grains with an average size around 10 µm and a few grains of intergranular Co_2_SnO_4_ spinel phase (grains with brightest contrast). Three areas of identical size (approximately 250 µm × 300 µm) were mapped: these are referred to as Areas 1, 2 and 3 in the following text. Pole-figures show random orientation of cassiterite grains in the microstructure [Fig. 1 ▸(*b*), for Area 1].

Grain boundaries in EBSD maps were identified by locating misorientations greater than 5° using the band contrast images as reference. It was found that a 10° threshold was not suitable for the investigated samples. The angle distribution of SnO_2_–SnO_2_ grain misorientation for Area 1 is presented in Fig. 1 ▸(*c*). The majority of grain contacts are random GBs with statistical distribution of angles as proposed by MacKenzie (1958 ▸) which is controlled by the point symmetry of cassiterite. The existence of frequent special GBs is revealed in the distribution of rotation angles, where the proportion of the rotation angles around ∼68° significantly exceeds those expected for a uniform orientation distribution. A close analysis of the disorientation along GBs related to this peak for Area 1 (67.8°±0.5°) results in an average rotation angle of 67.82±0.14 (*n* = 4582) with a rotation axis around [010]. The experimental closest parallel lattices correspond to (010)||(010) and [101]||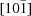 with an error of 0.1°. Given the small error in the following treatment, the TB is defined in terms of the exact relation of parallel lattices mentioned above.

In cassiterite with the rutile-type structure, TBs along {101} planes occur frequently, while twins on {301} are seldom (Nespolo & Souvignier, 2015 ▸; Hahn & Klapper, 2006 ▸; Ramdohr, 1969 ▸). The angle between [010] axes of the domains in twinned orientation may be used to distinguish between the two twin types. In {101} TBs, the angle between [010] axes of the twin domains is 67.8°, whereas in {301} TBs, the equivalent angle is 52.8° (Fig. S2). The misorientation angle for both types of TBs was mapped for all areas. The proportion of GBs corresponding to {101} TBs ranges from 17 to 20% of the total length of GBs, whereas the misorientation corresponding to 52.8° around [010] is below 0.2%. Of the 1275 grains in the three Areas, we detected only three contact twins with the contact angle corresponding to {301} TB. Although negligible, the existence of {301} TBs highlights the ability of EBSD mapping to detect special GBs in such extreme low proportion that would be extremely challenging for any other technique. The high frequency of {101} TBs observed by EBSD mapping confirms that this is the only relevant crystallographic contact in the samples as confirmed already in a previous study (Tominc *et al.*, 2018 ▸).

**Figure 2:**
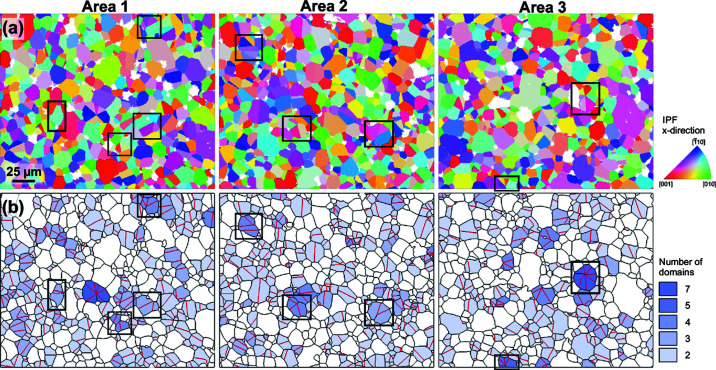
(*a*) Orientation maps of the three mapped Areas colored using the IPF color key (Nolze & Hielscher, 2016 ▸) and (*b*) general grain boundaries (black contours) and {101} twin boundaries (red) identified based on the misorientation analysis. Untwinned grains are shown in white, whereas twins with a different number of domains are highlighted in different shades of blue (see legend). The scale bar indicated in Area 1 is the same for all panels. [Crystallographic setting of nine multiply twinned grains (framed in black) will be analyzed in more detail in §3.2 and criteria for selection of these specific grains will also be given.]

Fig. 2 ▸(*a*) shows inverse pole figure (IPF)-colored maps of the three mapped regions, whereas Fig. 2 ▸(*b*) shows only highlighted boundaries between differently oriented cassiterite grains and domains. Black contours are general grain boundaries, whereas {101} twin boundaries are colored in red. A closer look at the images with boundary contours reveals that, in principle, three main types of grains with respect to the presence and number of {101} twin boundaries can be found in the microstructures. The first type corresponds to grains without TBs (untwinned grains), the second type are grains with only one TB referred to as contact twins in the following. The third and most interesting type corresponds to multiple twins (MTs) containing more than one TB per grain. Multiple twins composed of three to seven domains were found. In Fig. 2 ▸(*b*), grains with a different number of twin domains are highlighted with different shaded blue colors. Detection and shading of multiple twins with a different number of domains was done in *MTEX* by merging domains with a common twin boundary contact into a single grain.

Based on this analysis we quantitatively evaluated the number and the average equivalent circular diameter (ECD) for the different types of identified grains in the microstructures. The results for all three Areas are given in Table 1 ▸ (results for each Area separately are given in Table S1 in the supporting information) and show that untwinned grains represent about two thirds of all grains. Even though the majority of cassiterite grains are untwinned, contact and multiple twins appear regularly in the CoO and Nb_2_O_5_ co-doped SnO_2_-based ceramics and these grains are uniformly distributed in the microstructure. Contact twins are the most common type of twins representing about 27% of all grains, while cyclic twins are observed only in about 6% of all grains. The frequency of multiple twins decreases with the number of twin domains. In all three Areas we found only one multiply twinned grain with five twin domains and two multiply twinned grains with seven twin domains (see Table 1 ▸).

It is also interesting that average grain size seems to steadily increase with the number of twin domains. Untwinned grains have the smallest average equivalent diameter, contact twins are slightly larger and the multiple twins are the largest grains in the microstructure. The results also show that the size of multiple twins increases (linearly) with the number of twin domains. The two grains with seven domains are, for example, about 2.5 times larger than the average size of all grains. The different apparent ECD of multiple twins (especially those of more than three domains) suggests that there is a significant shape factor of the twinned grains with the direction of the common axis being the shortest. Because of the 2D sectioning effect of the cyclic twins, the proportion of contact twins is most likely overestimated.

### 3.2. EBSD analysis of multiple twins   

**Figure 3:**
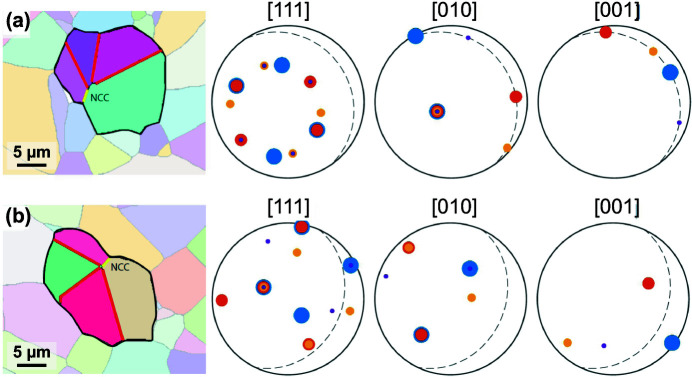
EBSD analysis of two cyclic twins composed of four domains. The {101} TBs are marked by red and the NCC in yellow. (*a*) The four domains have the [010] axis in common as indicated by overlapping of the [010] spots from all four domains. This confirms that this grain is a coplanar twin. (*b*) In this grain, the four domains have the [111] axis in common, therefore this is an alternating cyclic twin. Dashed lines indicate the common (111) and (010) planes for (*a*) and (*b*), respectively. Note that for (*a*) the *a*- and *c*-axes from all domains lie in the common (111) plane.

The crystallographic setting of cyclic twins was investigated in more detail by performing an automatic treatment of the EBSD data. In rutile-type minerals, the configuration of a cyclic twin is revealed from the crystallographic axis common to all domains. Fig. 3 ▸ shows EBSD images of two cyclic twins composed of four domains, which appear similar in the cross-section. Both grains contain three {101} twin boundaries and an additional NCC, similar to a general grain boundary. Angles between TBs in a cross-section depend on the orientation of the twinned grain, hence, the crystallographic setting of a cyclic twin cannot always be determined simply by measuring the angles between the TBs. EBSD analysis is therefore the only reliable tool to reveal the actual crystallographic configuration of a cyclic twin by allowing the consideration of all domains simultaneously. The analysis of the two grains depicted in Fig. 3 ▸ has shown that the four domains of the grain in Fig. 3 ▸(*a*) have the [010] axis in common, therefore this grain is a so-called coplanar cyclic twin. On the other hand, the domains of the grain shown in Fig. 3 ▸(*b*) have a common [111] zone axis indicating that this multiple twin has the so-called alternating configuration. This result confirmed that cyclic twins with two different crystallographic settings, alternating and coplanar, form in sintered SnO_2_ co-doped with Co- and Nb-oxide.

**Figure 4:**
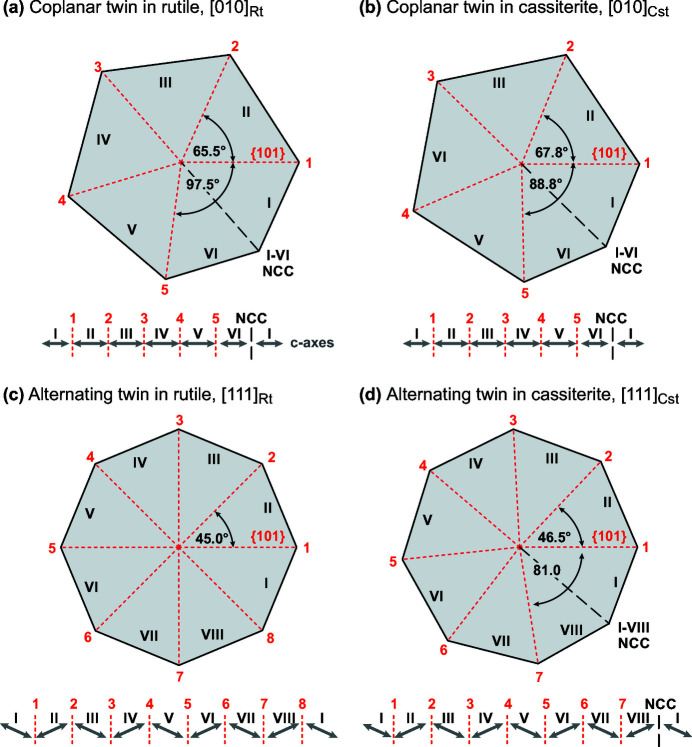
Schematic presentation of (theoretically) fully developed coplanar and alternating twins in rutile and cassiterite. (*a*,*b*) In coplanar twins, the maximum number of subsequent twin boundaries in both minerals is five and the cycle is closed by a non-crystallographic contact (NCC). The co-planar *c*-axes of twin domains are indicated by arrows below the projections along [010]. (*c*,*d*) In an alternating variant of cyclic twins, perfect eightlings can develop in rutile due to the almost exactly 45° angle between edge-on oriented {101} planes of the domains along the [111] zone axis; in cassiterite, however, the equivalent angle is 46.5°, allowing for a maximum of seven subsequent {101} twin boundaries and an additional NCC. In this type of twin, the *c*-axes of twin domains alternate up and down as shown below the projections along [111].

Cyclic twins are not characteristic for cassiterite, however, alternating and coplanar cyclic twins are common in rutile (Hahn & Klapper, 2006 ▸). The two minerals are isostructural and have similar unit-cell parameters (Table 2 ▸), therefore development of both types of cyclic twins can be anticipated in both minerals. Fig. 4 ▸ is a schematic presentation of theoretically fully developed coplanar and alternating twins in rutile and cassiterite.

The number of subsequent {101} twin boundaries depends on the crystallographic setting of a cyclic twin and the structural parameters of the mineral.

In coplanar cyclic twins, the limiting factor for the number of subsequent domains is the acute angle between two sets of {101} planes oriented edge-on along the [010] zone axis. In rutile, this angle is 65.5° [Fig. 4 ▸(*a*)], whereas it is slightly larger in cassiterite, 67.8° [Fig. 4 ▸(*b*)]. Theoretically, five subsequent {101} TBs fit into 360° in both minerals (*N* = 360°/α; rutile: 360/65.5 = 5.5; cassiterite: 360/67.8 = 5.3). Since *N* is not an integer in any of the phases, the cyclic twin is closed by an NCC between the first and the last domains. In coplanar cyclic twin configuration, the *c*-axes of twin domains lie in the same plane as schematically shown below the projections along [010]. The situation is more interesting in an alternating setting of cyclic twins, where the twin domains are oriented along the common [111] zone axis and the *c*-axes alternate ‘up and down’ around the common twin axis. In rutile, the acute angle between two sets of edge-on oriented {101} planes along the [111] zone axis is almost precisely 45°, meaning that exactly eight domains fit into 360° (360/45 = 8) [Fig. 4 ▸(*c*)]. In cassiterite, the equivalent angle is slightly larger, 46.5°; therefore only seven subsequent twin boundaries fit into 360° (360/46.5 = 7.7) and the gap between the first and the last domains would have to be closed by an NCC, similar as in coplanar twins [Fig. 4 ▸(*d*)].

Let us now inspect the cyclic twins found in our sample of Co- and Nb-oxide co-doped SnO_2_-based ceramics. EBSD analysis has shown the presence of cyclic twins composed of three and up to seven twin domains [Fig. 2 ▸(*b*)]. Cyclic twins with seven domains can only have the alternating configuration (see Fig. 4 ▸), whereas the configuration of those with three to six domains (five TBs + NCC) can only be unambiguously determined from EBSD data. In addition to twins of purely coplanar or alternating type, other twin combinations are possible. To facilitate detection of different types of multiple twins, automatic procedure for detection and classification of cyclic twins was developed in *MTEX* (the script is given in the supporting information).

**Figure 5:**
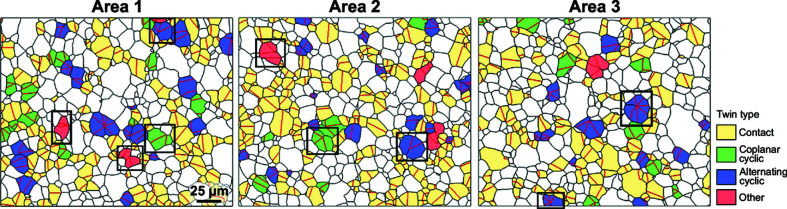
Determination of twin types using the automated detection procedure in *MTEX*.

The procedure includes recognition of domains sharing twin boundaries and merging these domains into a single grain [the result of this procedure is shown in Fig. 2 ▸(*b*)]. In the next step, the number of repeated/overlapping [111] and [010] directions is determined. Contact twins are composed by two domains that share two [010] and two [111] directions. For merged grains with three or more domains, the code identifies the number of coincident directions. If the number is equal to the number of domains per merged grain, the twin is classified as purely coplanar or alternating if the common axis is [010] or [111], respectively. Otherwise, the multiple twin is classified as ‘other’ type (see below). The results of the automated detection procedure of twin types for three mapped areas are shown in Fig. 5 ▸. All three types of multiple twins are randomly distributed in the sample indicating that the conditions for the formation of these twins occur uniformly throughout the sample during sintering. Quantitative analysis of the microstructures in terms of multiple twins is given in Table 3 ▸. The result shows that alternating twins are slightly more frequent than coplanar twins, while multiple twins of ‘other’ type are rare. The crystallographic setting of each multiple twins classified as ‘other’ type was analyzed separately. A careful individual analysis showed that most of these grains are combinations of an alternating or coplanar cyclic twin representing the core of the grain (usually with three domains) with an additional twin branch in random twin orientation with one of the domains of the cyclic twin. In addition, a grain with two parallel twin boundaries was found, which could be classified as a lamellar or polysynthetic twin. At this point it is also important to note that it cannot be excluded that some contact twins are indeed only sections of multiple twins, EBSD analyses are however inconclusive at classifying them as coplanar or alternating without performing 3D measurements (*e.g.* multiple sectioning).

To facilitate visualization of different types of multiple twins found in the sample, we reconstructed schematic 3D models of selected twins in *VESTA* (Momma & Izumi, 2011 ▸). Nine grains with different crystallographic settings and a different number of domains were selected in the three maps and these grains are framed in Figs. 2 ▸ and 5 ▸. Models were reconstructed based on quantitative orientation data from EBSD, where absolute orientation of each (indexed) crystal domain relative to the referential coordination system of the stage in the electron microscope is obtained.

**Figure 6:**
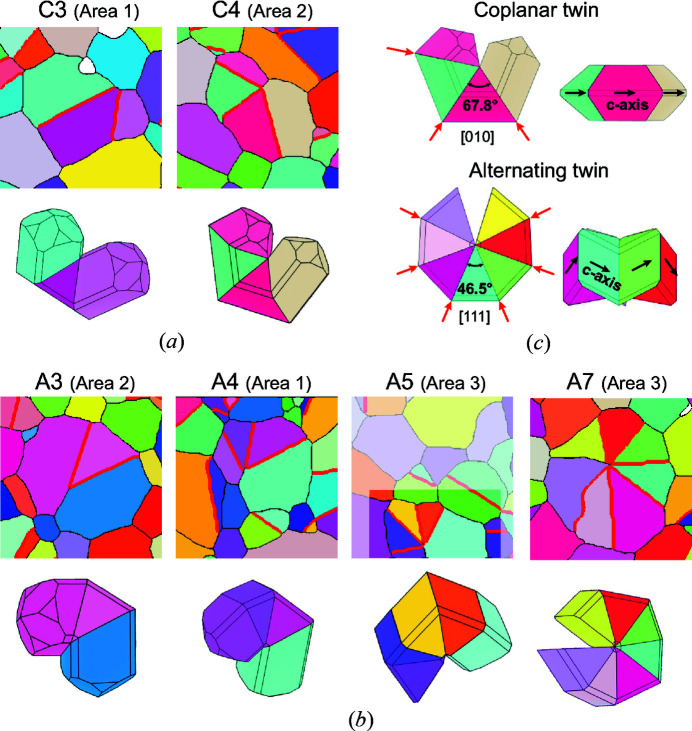
Schematic 3D models of coplanar and alternating twins with a different number of twin domains. The grains are framed in the maps shown in Figs. 2 ▸ and 5 ▸. (*a*) Coplanar twins with three (C3) and four (C4) domains. (*b*) Alternating twins with three (A3), four (A4), five (A5) and seven (A7) domains. (*c*) Models of a coplanar and an alternating twin oriented along the common twin axis and in side-view, where the difference in relative orientation of *c*-axes of the domains in each twin type is visible.

Although grains develop spherical morphologies without distinct crystal faces in the process of solid-state sintering, we reconstructed models as idealized faceted crystals surrounded by {110}, {010}, {101} and {111} faces for a more illustrative representation of crystallographic setting of the twins. Orientation of each 3D model shown in Figs. 6 ▸ and 7 ▸ corresponds exactly to the orientation of the sectioned grain and the colors of the twin domains correspond to the colors in the EBSD map. Fig. 6 ▸(*a*) shows coplanar twins with three and four domains (C3 and C4; where C stands for coplanar). In the three mapped areas we found several examples of coplanar twins with three and four domains, whereas coplanar twins with five or six domains as theoretically expected [see Fig. 4 ▸(*b*)] were not found. Alternating twins with three, four, five and seven domains (A3, A4, A5 and both A7 grains; A for alternating) are shown in Fig. 6 ▸(*b*). An alternating twin with six domains (A6) was not found, but it is likely that such grains exist since aggregates with five and seven domains occur. Cyclic twins with eight domains composed of seven subsequent TBs and an additional NCC as theoretically predicted [Fig. 4 ▸(*d*)] were not found either. Alternating twins with more than four domains are very rare (see Table 2 ▸), only one grain with five domains (Area 3) and two grains with seven domains (one in Area 1, another in Area 3) were found in areas mapped for this study. A coplanar and an alternating cyclic twin oriented along the twin axis and in side-view are shown in Fig. 6 ▸(*c*). Both types of cyclic twins have similar morphological features, for example, TBs always radiate from a common center (nucleus), which is positioned slightly eccentric relative to the grain section. Also, all cyclic twins in cassiterite are closed by an NCC, which is always shorter in comparison to the TBs in the twinned grain. These features are important for understanding the formation mechanism of cyclically twinned cassiterite.

**Figure 7:**
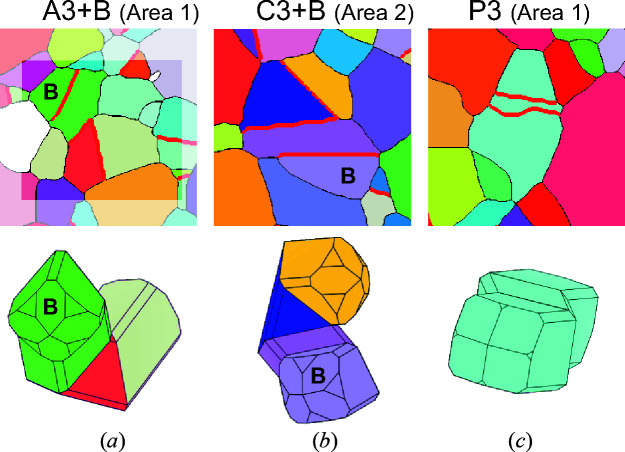
Three examples of multiple twins classified as ‘other’ type: (*a*) alternating twin with three domains and an additional branch (A3+B), (*b*) coplanar twin with three domains and an additional branch (C3+B) and (*c*) polysynthetic twin (P3) with two parallel twin boundaries.

Let us now inspect twinned grains of the ‘other’ type that were found in the sample and are colored in red in Fig. 5 ▸. These grains, shown in Fig. 7 ▸, are rare and, in reality, most of them can be described as a cyclic and an additional contact twin in random orientation (branch or leg). Figs. 7 ▸(*a*) and 7 ▸(*b*) show examples, where a typical coplanar or alternating cyclic twin is combined with an additional branch (B) in twin orientation with one of the domains of the cyclic twin. Another type of multiple twins is polysynthetic (or lamellar) twins [Fig. 7 ▸(*c*)]. This is the least frequently detected multiple twin type by SEM/EBSD and only one such grain was found. Models of cassiterite twins constructed in *VESTA* (.mp4 video files), shown in Figs. 6 ▸ and 7 ▸, are provided in the supporting information.

## 4. Discussion   

### 4.1. Formation of cyclic twins via nucleation twinning   

In this work we used EBSD for the analysis of multiple twins that occur in SnO_2_-based ceramics sintered with small additions of Co- and Nb-oxides. We detected two main types of multiple twin, the first are coplanar cyclic twins with a [010] common axis and composed of three or four domains and the second are alternating cyclic twins with a [111] common axis and three to seven twin domains. Even though only about 6% of all grains are cyclic twins, they form regularly in SnO_2_ co-doped with Co-oxide and Nb-oxide. This percentage is, however, a minimum estimated due to the lower likelihood during sectioning. Cyclic twins do not form in undoped SnO_2_ nor in SnO_2_ sintered only with the addition of Co-oxide, where only few contact twins were observed (Tominc *et al.*, 2018 ▸). Recently, cyclic twins were found also in Co- and Ta-oxide co-doped SnO_2_ (Tominc *et al.*, 2020 ▸). This implies that co-doping with the two aliovalent oxides plays the key role in the formation of cyclic twins, however, the exact formation mechanism remains unclear. The proposed mechanisms for the formation of cyclically twinned particles in different materials and under various crystal growth conditions include twinning during nucleation, growth-mediated formation and oriented attachment (Senechal, 1980 ▸; Hofmeister, 2004 ▸; Hahn & Klapper, 2006 ▸; Nespolo & Ferraris, 2004 ▸; Song *et al.*, 2019 ▸).

Coplanar and alternating cyclic cassiterite twins are crystallographically analogous to rutile sixlings and eightlings found at Magnet Cove (Arkansas, USA) (Howard, 1999 ▸; Hahn & Klapper, 2006 ▸). It has been suggested that the rutile eight­lings are nucleation twins, meaning that they start to grow from a nucleus (Hahn & Klapper, 2006 ▸). Erickson & Blade (1963 ▸) studied the geochemistry and petrology of the alkaline igneous complex at Magnet Cove and found that cyclic twins of rutile contain unusually high concentrations of Nb and Fe, indicating a possible role of these (aliovalent) elements in the nucleation of cyclic twins of rutile. Nucleation twinning theory for the formation of cyclic twins in cassiterite is additionally supported by morphological characteristics of coplanar and alternating twins, where all TBs always radiate from the common point or the twin nucleus. Since cyclic twins only form in co-doped SnO_2_, it might be assumed that nucleation starts on a pre-existing Co-Nb-oxide which is structurally related to cassiterite. Formation of cyclic twins with purely coplanar or alternating setting would also suggest that nuclei with different structure are involved in their formation. According to Senechal (1980 ▸), twinning by nucleation starts on a nucleus with different or even non-crystallographic symmetry. In the formation of ZnO tetrapods, for example, where two neighboring legs are in twin orientation, wurtzite (*hcp*) ZnO nucleates on pre-existing sphalerite ZnO (*ccp*) nucleus with tetrahedral morphology (Ding *et al.*, 2007 ▸). The nucleation twinning theory for the formation of cyclic twins assumes that the critical size of the nucleus is in the nano-range, its structure is metastable and therefore it probably recrystallizes during the growth of the twinned crystal.

In addition to the nucleation on a precursor phase, the formation of twin boundaries might be additionally enhanced by local strain in the crystal structure due to the incorporation of dopants. In our previous study we showed that the growth of SnO_2_ grains is governed by diffusion processes that involve incorporation of up to a few atomic percent of both aliovalent dopants (Co^2+^ and Nb^5+^) to regular Sn sites in approximately 1:4 ratio and that the charge inside the SnO_2_ grains is balanced by the formation of Sn^2+^ (Tominc *et al.*, 2018 ▸). This situation is similar to the one observed for Fe^3+^/Nb^5+^ co-doped TiO_2_-based ceramics, where the dopants are incorporated in the rutile matrix substituting regular Ti sites and, concomitantly, the Ti ions are partially reduced to Ti^3+^ (Nachaithong *et al.*, 2018 ▸). The presence of Sn^2+^ and Ti^3+^ with larger ionic radii inside the cassiterite or rutile structure, respectively, may cause local strain and trigger the formation of twin boundaries.

### 4.2. The role of TBs in the growth of cyclic twins   

After the nucleation of a cyclic twin, further growth of the composite (twinned) grain depends also on the growth rate of contact {101} twins between the neighboring domains. In cyclic twins of cassiterite it is typically observed that the {101} TBs are much longer than the NCC, which closes up the cyclic twin. In addition, quantitative analysis of the microstructure has shown that contact twins are larger than untwinned grains, whereas the size of multiple twins increases with the number of twin domains. Both alternating twins with seven domains are about 2.5 times larger than the average size of all grains (see Table 1 ▸). This indicates that the formation of twin boundaries has an enhancing effect on the cassiterite grain growth. In our previous work we analyzed the local structure and chemistry of the twin boundaries and found only non-uniform segregation of Co and Nb near the TB plane (Tominc *et al.*, 2018 ▸). This confirmed that {101} TBs in cassiterite are not chemically induced growth-type planar defects, where the dopant that triggers the formation of the defect is incorporated into the contact plane (interface) in a periodic (ordered) manner. Crystals with chemically induced defects exhibit exaggerated grain growth along the interface and develop distinctly anisotropic morphologies. An example of chemically induced TBs are {111} twins in MgAl_2_O_4_ spinel, which form as a result of beryllium incorporation into the twin boundary structure (Drev *et al.*, 2013 ▸). In cassiterite, however, the apparent faster growth of the {101} twin boundaries might be related to the stability of the {101} twin stacking. Lee *et al.* (1993 ▸) have calculated the energy of {101} and {301} twin boundaries in rutile and cassiterite with different local structure. They found that {101} pseudo twin configuration is the one with the lowest energy and that it is characterized by the ½〈111〉 in-plane translation and mirror symmetry of the metal sublattice across the twin boundary. It is possible, however, that the presence of dopants further stabilizes the twin contact. This is additionally supported by Kawamura *et al.* (1999 ▸), who showed that the addition of pentavalent dopants like Nb_2_O_5_ and Ta_2_O_5_ trigger abundant formation of contact twins in cassiterite grown in Cu_2_O flux. The influence of TB formation on the growth of cassiterite grains implies that understanding the nucleation mechanism of contact and multiple twins may provide an efficient tool for controlling grain growth and microstructure development in functional SnO_2_-based ceramics.

## 5. Conclusions   

We found that the microstructure of SnO_2_-based ceramics sintered with small additions of Co- and Nb-oxides consists of untwinned grains, contact twins and a small fraction of multiple, mostly cyclic twins with coplanar and alternating morphology. Since multiple twins are practically indistinguishable in SEM cross-sections in terms of their crystallographic setting, we used EBSD orientation mapping to determine the common axis of all domains to distinguish between multiple twins with different morphologies. We developed a protocol in *MTEX* for an automated detection and identification of twinned grains regardless the number of twin domains. The approach is generally applicable to other systems where twins with different crystallographic configurations are found.

Statistical evaluation of the microstructures in terms of grains type (untwinned grains, contact twins, coplanar cyclic twins, alternating cyclic twins and ‘other’ multiple twins) and grains size has shown that twinned grains are larger in comparison to untwinned grains. Also, the size of twins increases linearly with the number of twin domains due to the faster growth along the {101} TB planes. This implies TBs have an enhancing effect on SnO_2_ grain growth and that control over the formation of TBs in cassiterite-based ceramics may represent a powerful mechanism for tailoring microstructures of ceramics with targeted grain size, which is vital for the production of ceramics with controlled functional properties. In order to achieve this goal, the formation mechanism of TBs (contact and cyclic) will have to be further studied. Current results suggest that the formation of twins is triggered by nucleation on a Co–Nb-containing precursor phase. The role of locally induced strain due to the incorporation of dopants into the cassiterite crystal must also be considered.

## Supplementary Material

Additional figures, schemes, tables and MTEX scripts. DOI: 10.1107/S2052520620010264/yh5004sup1.pdf

Model of cassiterite twins constructed in VESTA: A3+B Area 1. DOI: 10.1107/S2052520620010264/yh5004sup2.mp4

Model of cassiterite twins constructed in VESTA: A3 Area 2. DOI: 10.1107/S2052520620010264/yh5004sup3.mp4

Model of cassiterite twins constructed in VESTA: A4 Area 1. DOI: 10.1107/S2052520620010264/yh5004sup4.mp4

Model of cassiterite twins constructed in VESTA: A5 Area 3. DOI: 10.1107/S2052520620010264/yh5004sup5.mp4

Model of cassiterite twins constructed in VESTA: A7 Area 3. DOI: 10.1107/S2052520620010264/yh5004sup6.mp4

Model of cassiterite twins constructed in VESTA: C3+B Area 2. DOI: 10.1107/S2052520620010264/yh5004sup7.mp4

Model of cassiterite twins constructed in VESTA: C3 Area 1. DOI: 10.1107/S2052520620010264/yh5004sup8.mp4

Model of cassiterite twins constructed in VESTA: C4 Area 2. DOI: 10.1107/S2052520620010264/yh5004sup9.mp4

Model of cassiterite twins constructed in VESTA: P3 Area 1. DOI: 10.1107/S2052520620010264/yh5004sup10.mp4

## Figures and Tables

**Table 1:** Quantitative analysis of ceramics microstructure TB = twin boundaries, *N* = number of grains, ECD = equivalent circular diameter, MT = multiple twins (coplanar, alternating and branched).

	Absolute values			Relative to all grains	
	*N*	ECD (µm)	Std	*N* (%)	ECD (%)
All grains	1275	10.6	2.8	100	100
Untwinned grains	849	9.8	2.8	66.6	92
Contact twins	345	11.5	2.2	27.1	108
MT - all	96			6.3	
MT - 3 domains	62	14.3	2.2	4.9	137
MT - 4 domains	16	17.3	2.5	1.3	163
MT - 5 domains	1	16.4†		0.1	154†
MT - 6 domains	Not found				
MT - 7 domains	2	27.0	0.1‡	0.2	254

† Underestimated because the grain is partially cut off at the bottom edge of Area 3 map.

‡ Based on only two grains.

**Table 2:** Cell parameters of rutile and cassiterite (space group *P*4_2_/*mnm*, No. 136) and acute angles between two sets of {101} planes oriented edge-on in any of the 〈010〉 or 〈111〉 zone axes

Rutile, TiO_2_ (Howard *et al.*, 1991 ▸)	Cassiterite, SnO_2_ (Bolzan *et al.*, 1997 ▸)
*a* = 0.45937 nm	a = 0.47374 nm
*c* = 0.29587 nm	c = 0.31864 nm
〈010〉: ∠ {101} = 65.5°	[010]: ∠ {101} = 67.8°
〈111〉: ∠ {101} = 45.0°	[111]: ∠ {101} = 46.5°

**Table 3:** Occurrence of different types of multiple twins given as fraction of all grains (1275, absolute) and as a fraction of all multiple twins (77, relative)

	Absolute (%)	Relative (%)
Coplanar	2.2	36.4
Alternating	3.3	54.5
Other	0.5	9.1
