# Supplementary material for: Twinning in SnO2-based ceramics doped with CoO- and Nb2O5: morphology of multiple twins revealed by electron backscatter diffraction
Source: Acta Crystallogr B Struct Sci Cryst Eng Mater. 2020 Sep 15;76(Pt 5):875–83. doi: 10.1107/S2052520620010264 (PMC7535064; doi:10.1107/S2052520620010264)
Supplement: Supplementary file 1 [file b-76-00875-sup1.pdf]

STRUCTURAL SCIENCE
CRYSTAL ENGINEERING
MATERIALS

Volume 76 (2020)

Supporting information for article:

Twinning in SnO2-based ceramics doped with CoO- and Nb2O5: morphology of multiple twins revealed by electron backscatter diffraction

José Alberto Padrón-Navarta, Fabrice Barou and Nina Daneu

1. Band contrast images of Areas 2 and 3

In addition to Area 1, for which the band contrast image is shown in the manuscript, another two areas (Area 2 and 3) with identical dimensions were mapped. Figure S1 shows band contrast images for these two areas. The images are similar to the microstructure of Area 1, both show typical microstructure of polycrystalline untextured ceramics, composed of pseudo spherical grains with average size of around 10 microns. In addition, the samples contain few grains of secondary spinel phase ($CoSn_2O_4$) which have brighter contrast and some pores. Boundaries between grains and twin domains are exposed as result of thermal etching. Grain boundaries are typically curved, while twin boundaries are straight and intersect of divide the parent grain to domains.

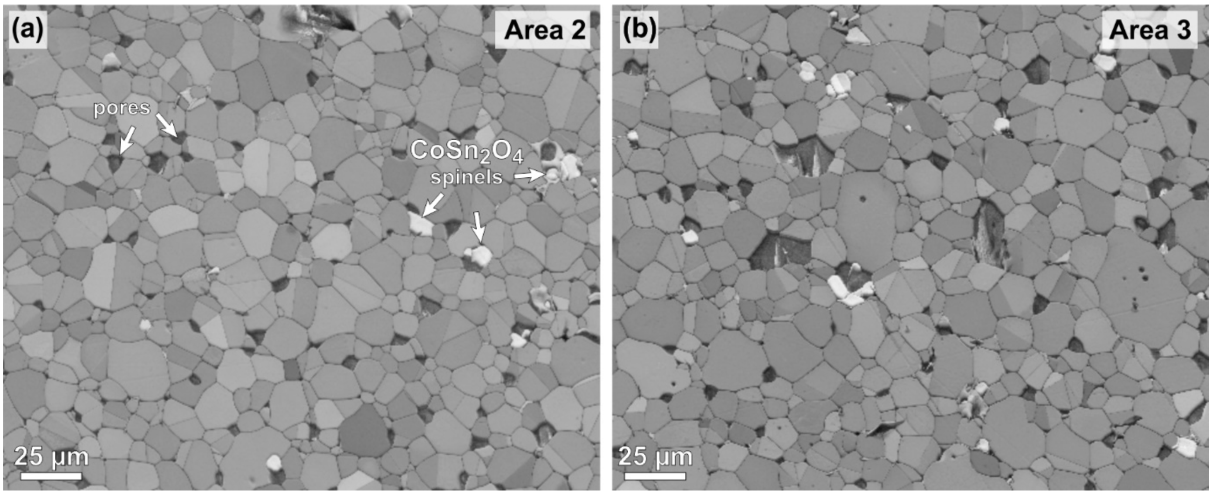

Figure S1: Band contrast images of (a) Area 2 and (b) Area 3. In addition to cassiterite grains as the major phase, the sample contains few grains of secondary $CoSn_2O_4$ spinel phase and some pores.

2. Determination of {101} and {301} twins in cassiterite with EBSD

In EBSD, relative orientation between grains or crystal domains is determined from the angle between two low-index crystallographic directions. In polycrystalline ceramics, where the grains are randomly oriented, grain boundaries have statistical distribution of angles MacKenzie (1958). In contrast, for crystallographic contacts like twin boundaries, the angle is constant. In cassiterite, two types of crystallographic contacts are possible: twin boundaries on {101} or {301} planes. EBSD can distinguish between the two twins based on the angle between [001] directions of cassiterite domains as shown in Figure S1.

In the case of {101} twin (Fig. S2a) the angle between the [001] ayes is 67.8°. In the case the {301} twin, the relative rotation of two crystal lattices around the [010] direction is 127.2°. In EBSD, however, the supplementary angle (180° - 127.2°) is measured, therefore the search for {301} twins in EBSD is performed by searching for the cassiterite domains with apparent 52.8° angle between their [001] axes.

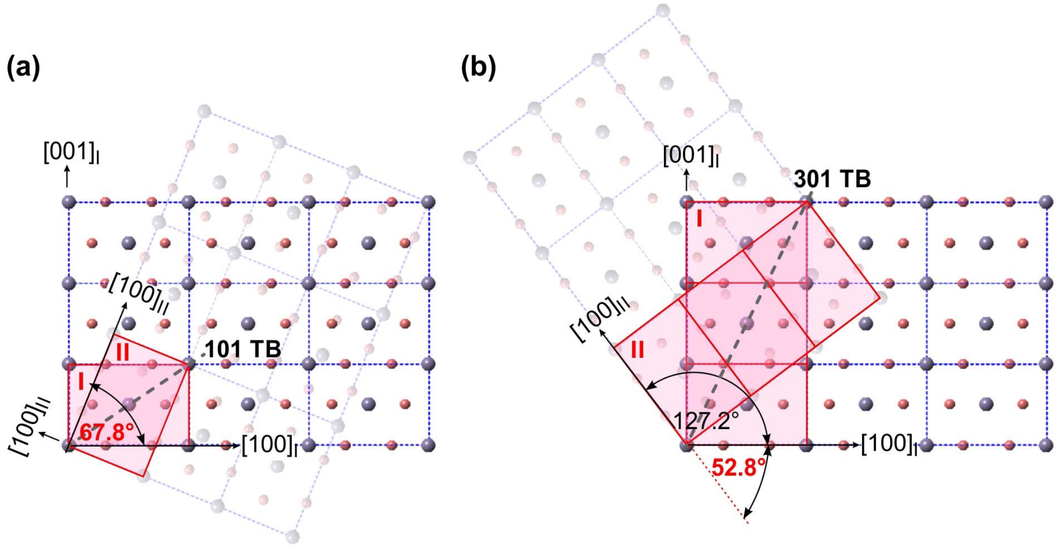

Figure S1: Schematic presentation of the angle between [001] directions of two domains in (a) {101} and (b) {301} twin orientation.

3. Quantitative analysis of microstructures (Areas 1, 2 and 3)

In the manuscript we show three mapped Areas, recorded on the same sample. Each Area was analyzed separately and the results are given in Table S1 (size and type of grains - untwinned, contact and multiple twins) and S2 (analysis of multiple twin types).

Table S1: Quantitative analysis of ceramics microstructure. TB = twin boundaries, N = number of grains, ED = equivalent diameter, MT = Multiple twins (coplanar, alternating and branched)

	Area 1			Area 2			Area 3		
	N	ED, μm	Std, μm	N	ED, μm	Std, μm	N	ED, μm	Std, μm
All grains	407	10.9	2.8	470	10.1	2.6	398	11.0	2.9
Untwinned grains	264	10.1	2.9	305	9.3	2.7	279	10.2	2.9
Contact twins	110	11.4	2.2	138	11.1	2.2	97	12.3	2.4
Multiple twins	33			27			22		
MT - 3 domains	27	15.5	2.1	22	13.4	2.3	14	13.5	2.4
MT - 4 domains	5	15.9	2.1	5	17.4	3.1	6	18.4	2.4
MT - 5 domains	-			-			1	16.4	
MT - 6 domains	-			-			-		
MT - 7 domains	1	27.1					1	26.9	
Relative proportions (relative to All grains), %									
Untwinned grains	65	92		65	92		70	93	
Contact twins	27	105		29	110		24	112	
Multiple twins	8			6			6		
MT - 3 domains	7	142		5	132		4	123	
MT - 4 domains	1	145		1	172		2	168	
MT - 5 domains							0.25	150	
MT - 6 domains									
MT - 7 domains	0.25	248		0.25			0.25	245	

Table S2: Relative occurrence of different types of multiple twins given separately for the three mapped areas.

	Area 1, %		Area 2, %		Area 3, %	
	absolute	relative	absolute	relative	absolute	relative
Coplanar	3.2	41.9	1.3	24.0	2.3	42.9
Alternating	3.7	48.4	3.4	64.0	2.8	52.4
Other	0.7	9.7	0.6	12.0	0.3	4.8

4. MTEX script for identification of multiple cyclic twins, their type and statistical treatment of the data

```
CS = {'notIndexed',...
  crystalSymmetry('4/mmm', [4.7374 4.7374 3.1864], 'mineral', 'Cassiterite', 'color', 'light blue'),...
  crystalSymmetry('m-3m', [8.6461 8.6461 8.6461], 'mineral', 'Co2SnO4', 'color', 'light green')};

% Plotting convention
setMTEXpref('xAxisDirection','east');
setMTEXpref('zAxisDirection','intoPlane');

% Path to files
pname = '/EBSD_maps';
fname = [pname '/Cassiterite-Area1.cpr'];
ebsd = EBSD.load(fname,CS,'interface','crc', 'convertEuler2SpatialReferenceFrame');
CS = ebsd('Cassiterite').CS;

% Segment grains
[grains,ebsd.grainId,ebsd.mis2mean] = calcGrains(ebsd('indexed'),'angle',5*degree);
ebsd(grains(grains.grainSize<=5)) = [];
ebsd('Co2SnO4') = [];
[grains,ebsd.grainId,ebsd.mis2mean] = calcGrains(ebsd('indexed'),'angle',5*degree);

% Smooth them
grains = grains.smooth(3);

% Grain boundaries
gB = grains('Cassiterite').boundary;
gB_CassCass = gB('Cassiterite','Cassiterite');

% Define a twinning misorientation
twinning = orientation.map(Miller(0,1,0,CS),Miller(0,1,0,CS),...
  Miller(1,0,1,CS),Miller(1,0,-1,CS));

% Restrict to twinnings with threshold 3 degree
isTwinning = angle(gB_CassCass.misorientation,twinning) < 2*degree;
twinBoundary = gB_CassCass(isTwinning);

[mergedGrains,parentId] = merge(grains,twinBoundary);

% Algorithm to automatically classify cyclic and complex twins
L = [parentId grains.id]; % index of parent grain and original grains.id
[C ia ic] = unique(L(:,1), 'stable'); % find repeated values (unique values)...
h = accumarray(ic, 1); % counts number of repetitions

% Augmented L with the number of times each parent Id repeats
L = [L h(ic(1:length(L(:,1))))];

N_domains = L;
[list_domains ia ic] = unique(N_domains(:,1));

m111 = Miller(1,1,1,'uvw',CS);
m010 = Miller(0,1,0,'uvw',CS);
```

```
m001 = Miller(0,0,1,'uvw',CS);

n_coincident = zeros(length(list_domains),2);
for i=1:length(list_domains)
    % matrix containing all xyz vector 3d per twinned parent grain
    R_111 =
unique(grains(N_domains(N_domains(:,1)==list_domains(i),2)).meanOrientation*m111.symmetrise).
xyz;
    [C,ia,ic] = uniquetol(R_111,0.02, 'ByRows', true);
    h = accumarray(ic, 1);
    n_coincident(i,1) = max(h);
    R_010 =
unique(grains(N_domains(N_domains(:,1)==list_domains(i),2)).meanOrientation*m010.symmetrise).
xyz;
    [C,ia,ic] = uniquetol(R_010,0.02, 'ByRows', true);
    h = accumarray(ic, 1);
    n_coincident(i,2) = max(h);
end

[list_domains ia ic] = unique(N_domains(:,1));
class = [list_domains N_domains(ia,3) n_coincident];

type = zeros(length(class(:,1)),1);
for i = 1:length(class(:,1))
    if class(i,3) == class(i,2) & class(i,4) < class(i,2)
        type(i) = 1;
    elseif class(i,4) == class(i,2) & class(i,3) < class(i,2)
        type(i) = 2;
    else
        type(i) = 0;
    end
end
FINAL = [class type]


%% Statistics
ED = zeros(8,3);
ED(1,1) = mergedGrains.length;
ED(1,2) = 2*mean(mergedGrains.equivalentRadius);
ED(1,3) = std(mergedGrains.equivalentRadius);
for i = 2:8
ED(i,1) = mergedGrains(FINAL(FINAL(:,2)==i-1)).length;
ED(i,2) = 2*mean(mergedGrains(FINAL(FINAL(:,2)==i-1)).equivalentRadius);
ED(i,3) = std(mergedGrains(FINAL(FINAL(:,2)==i-1)).equivalentRadius);
end

labels = ["All grains"; "1 domain"; "2 domains"; "3 domains"; "4 domains"; "5 domains"; "6
domains";...
    "7 domains"];
table(labels, ED)

alternating = length(FINAL(FINAL(:,2)>2 & FINAL(:,5)==1));
coplanar = length(FINAL(FINAL(:,2)>2 & FINAL(:,5)==2));
other = length(FINAL(FINAL(:,2)>2 & FINAL(:,5)==0));
```

```
labels2 = ["Alternating"; "Coplanar"; "Other"];
table(labels2, [alternating; coplanar; other])
```

5. MTEX script for the conversion of EBSD data to vectors for Vesta

```
%%  Convert for Vesta (Z = projection vector; Y = upward vector)
% where eulers is a vector containing eulers angles
% the sign of the z-vector and y-vector depends on the reference frame of the stage, in the present
study
% plotting convention
setMTEXpref('xAxisDirection','east');
setMTEXpref('zAxisDirection','intoPlane');
ss = specimenSymmetry('1');
o = orientation('Euler',eulers,ebsd('Cassiterite').CS,ss);
Z = inv(o) * -zvector;
Miller(Z,'uvw')
Y = inv(o) * -yvector;
Miller(Y,'hkl')
```

6. 3D models of twins

3D models of selected twins (*see* Figs. 2, 5, 6 and 7 in the main paper) were constructed in Vesta (Momma & Izumi, 2011). For the supporting information we recorded short video clips in which the models are rotated in space for their better visualization of the twins:

1. C3-area1.mp4 (Fig. 6a)
2. C4-area2.mp4 (Fig. 6a)
3. A3-area2.mp4 (Fig. 6b)
4. A4-area1.mp4 (Fig. 6b)
5. A5-area3.mp4 (Fig. 6b)
6. A7-area3.mp4 (Fig. 6b)
7. C3+B-area2.mp4 (Fig. 7a)
8. A3+B-area1.mp4 (Fig. 7b)
9. P3-area1.mp4 (Fig. 7c)
